# Reply: Comment on ‘*MicroRNA-214* suppresses growth, migration and invasion through a novel target, high mobility group AT-hook 1, in human cervical and colorectal cancer cells’

**DOI:** 10.1038/bjc.2016.410

**Published:** 2016-12-20

**Authors:** Karthik Subramanian Chandrasekaran, Anusha Sathyanarayanan, Devarajan Karunagaran

**Affiliations:** 1 Department of Biotechnology, Bhupat and Jyoti Mehta School of Biosciences, Indian Institute of Technology Madras, Chennai, Tamil Nadu 600036, India

**Sir,**

The authors would like to acknowledge the time and effort spent in critical assessment of their work (Chandrasekaran *et al*, 2016) by Cristóbal *et al.* Since they have pointed out that the effect on the HMGA1 protein expression is minimal, the authors draw their attention to these data:

1. The ectopic expression of *miR-214* reduced mRNA levels of *HMGA1* by 15–30% (Figure 2B) and *HMGA1* 3′UTR-regulated luciferase activity by 25–33% (Figure 2E) in SW480 and SW620 cells, respectively.
2. The effect of ectopic expression of *miR-214* on HMGA1 protein levels in these cell lines is quite consistent and reproducible.
3. Suppressing the endogenous expression of *miR-214* has profound effect on *HMGA1* expression at the protein level (increased HMGA1 by 40–70%) in both these cell lines (Figure 5A). This clearly points out the ability of endogenous *miR-214* to suppress *HMGA1* expression.

These reasons are enough to claim that *miR-214* targets *HMGA1* and downregulates its expression. The complexity involved in several miRNAs regulating the expression of a single target spatially and temporally has been discussed in the paper and stated in the query itself. Decoding the entire regulatory miRNA network of *HMGA1* was not within the scope of this study.

Cristóbal *et al* have pointed out that the anti-tumourigenic effects of *miR-214* might be mediated through the Wnt/*β*-catenin pathway and not by *HMGA1*. Once again, attention is drawn to these:

1. The effects of ectopic expression of *miR-214* (Figures 3A–C) and suppression of expression of *HMGA1* (Figures 4A–C) on proliferation, migration and invasion of SW480 and SW620 cells are very much comparable.
2. The effects of ectopically co-expressing *miR-214* and *HMGA1* (as suggested in the query) were addressed (Figure 6), and the conclusions are not likely to change by ectopically expressing *miR-214* or suppressing HMGA1 expression together.
3. The reference cited to show the ability of HMGA1 to positively regulate the Wnt-*β*-catenin pathway in the query also suggests that *miR-214* could directly suppress *β*-catenin, downregulating the Wnt pathway through negative regulation of *HMGA1*. In addition, this cannot be directly extended to CRC since the referred studies were conducted in hepatic cancer cells and will need experimental validation in CRC cells, which are known to exhibit constitutive Wnt signalling.

In summary, since *miR-214* does indeed target *HMGA1* and downregulates its expression, and since *HMGA1* certainly behaves as an oncogene in CRC cells, part of the tumour-suppressive functions of *miR-214* may occur through suppression of *HMGA1* expression. The functional relevance of *miR-214* targeting *HMGA1* could be inferred from the fact that *miR-214* is still able to exert its tumour-suppressing function in the presence of ectopically expressed *HMGA1*.

Further, the study focused on ascertaining whether an inverse correlation exists between the expression levels of *miR-214* and *HMGA1*. Since a study on 20 pairs (normal and tumour) of tissues could be considered insufficient to obtain conclusiveness, a much larger population (322 samples from TCGA) was analysed; the results from both the analyses reinforced each other and evidently showed the inverse correlation in the expression levels of *miR-214* and *HMGA1* (Figure 1).

The authors agree with Cristóbal *et al* that protein-mRNA correlation (with respect to *HMGA1* expression) would help in concluding about the status of *HMGA1* in CRC and would strengthen the conclusions. The contradiction observed in *HMGA1* status in CRC tissues (Liang *et al*, 2013) could be attributed to differences in the genetic make-up of the populations studied, and their results are contraindicated by the data on *HMGA1* expression in CRC obtained from TCGA. In conclusion, the authors would like to reiterate that the study does not discount the roles that may be played by other miRNAs that may target *HMGA1* or other targets of *miR-214*, and this is discussed in the paper. This is why, in quite a few relevant instances, it is asserted that *miR-214*, in part, suppresses growth and motility in cancer cells by downregulating *HMGA1*, as it is intuitively recognised that further studies are most certainly required to unravel the outcome of a miRNA-regulatory network on the behaviour of *HMGA1* in CRC and other cancers.
